# Transforming Growth Factor-β Drives the Transendothelial Migration of Hepatocellular Carcinoma Cells

**DOI:** 10.3390/ijms18102119

**Published:** 2017-10-10

**Authors:** Petra Koudelkova, Victor Costina, Gerhard Weber, Steven Dooley, Peter Findeisen, Peter Winter, Rahul Agarwal, Karin Schlangen, Wolfgang Mikulits

**Affiliations:** 1 Department of Medicine I, Division: Institute of Cancer Research, Comprehensive Cancer Center, Medical University of Vienna, 1090 Vienna, Austria; koudelkova.petra@yahoo.com (P.K.); gerhard.weber@meduniwien.ac.at (G.W.); 2 Institute for Clinical Chemistry, Medical Faculty Mannheim, University of Heidelberg, University Hospital Mannheim, 68167 Mannheim, Germany; Victor.Costina@umm.de (V.C.); Peter.Findeisen@umm.de (P.F.); 3 Molecular Hepatology Section, Department of Medicine II, Medical Faculty Mannheim, University of Heidelberg Mannheim, 68167 Mannheim, Germany; steven.dooley@medma.uni-heidelberg.de; 4 GenXPro GmbH, 60438 Frankfurt am Main, Germany; pwinter@genxpro.de (P.W.); raag@genxpro.de (R.A.); 5 Center for Medical Statistics, Informatics and Intelligent Systems, Medical University of Vienna, 1090 Vienna, Austria; schlangen@spengergasse.at

**Keywords:** hepatocellular carcinoma, TGF-β, transendothelial migration, SILAC, proteomics, bioinformatics

## Abstract

The entry of malignant hepatocytes into blood vessels is a key step in the dissemination and metastasis of hepatocellular carcinoma (HCC). The identification of molecular mechanisms involved in the transmigration of malignant hepatocytes through the endothelial barrier is of high relevance for therapeutic intervention and metastasis prevention. In this study, we employed a model of hepatocellular transmigration that mimics vascular invasion using hepatic sinusoidal endothelial cells and malignant hepatocytes evincing a mesenchymal-like, invasive phenotype by transforming growth factor (TGF)-β. Labelling of respective cell populations with various stable isotopes and subsequent mass spectrometry analyses allowed the “real-time” detection of molecular changes in both transmigrating hepatocytes and endothelial cells. Interestingly, the proteome profiling revealed 36 and 559 regulated proteins in hepatocytes and endothelial cells, respectively, indicating significant changes during active transmigration that mostly depends on cell–cell interaction rather than on TGF-β alone. Importantly, matching these in vitro findings with HCC patient data revealed a panel of common molecular alterations including peroxiredoxin-3, epoxide hydrolase, transgelin-2 and collectin 12 that are clinically relevant for the patient’s survival. We conclude that hepatocellular plasticity induced by TGF-β is crucially involved in blood vessel invasion of HCC cells.

## 1. Introduction

Hepatocellular carcinoma (HCC) is the sixth most common malignancy worldwide and ranks third in mortality among cancer [1,2]. HCC frequently arises from long-term inflammatory conditions and is mostly diagnosed at advanced stages resulting in poor prognosis [3]. The development of HCC is classified by the Barcelona Clinic Liver Cancer (BCLC) staging with a link to therapeutic options [4]. At very early (BCLC-0) and early (BCLC-A) stages displaying a uni-nodular disease, tumor resection or liver transplantation provide curative therapeutic options [5]. At advanced stages of HCC with multiple nodules (BCLC-B) including vessel invasion (BCLC-C) and intra- and extrahepatic metastasis (BCLC-C), however, treatment remains solely palliative with five-year overall survival rates below 10% (Cancer Facts & Figures 2016; Atlanta: American Cancer Society).

One crucial event that strongly worsens the patient’s prognosis is the entry of HCC cells into the vasculature and the subsequent dissemination to intra- or extrahepatic metastatic sites [6]. While intrahepatic metastasis is frequently observed at late HCC stages, extrahepatic metastasis to e.g., the lung occurs in only 10–20% of all HCC patients. For a successful metastatic spread, cancer cells must proceed through several steps to initiate colonization at distal sites [7]. In this scenario, neoplastic epithelial hepatocytes are considered to undergo reprogramming by transforming into a migratory, mesenchymal-like phenotype during a process called epithelial-mesenchymal transition (EMT) [8,9]. Multiple reports provide evidence that EMT-transformed hepatocytes invade the surrounding stromal microenvironment and move towards endothelial cells to cross the barrier and get entry into the circulation for further dissemination. Thereby, invading hepatocytes interact with endothelial cells and transmigrate through the endothelial layer in a process termed intravasation, which is still poorly understood in HCC.

A solid tumor is a hostile place, which already at a diameter of 2 mm, suffers from low oxygen and nutritional supply [10]. To overcome this consistent survival pressure, cancer cells produce endogenous growth factors that stimulate angiogenesis and enhance tumor cell survival through the formation of new blood vessels [11]. It is commonly believed that excessive proliferation of endothelial cells in tumor neo-angiogenesis leads to fragile and leaky vessels which cannot properly maintain their barrier function [12]. However, recent findings suggest that intravasation does not appear randomly. Instead, intravasation seems to be a well-orchestrated event, in which both cancer and endothelial cells mediate the transmigration process. In vitro studies showed that the interaction between cancer cells and endothelial cells may lead to changes in biomechanical properties of the endothelial barrier, thereby reducing its stiffness [13]. Interestingly, migrating cancer cells actively force their way through the endothelium as it has been shown for cancer cells with overexpression and activation of the epidermal growth factor receptor, which induces a neutrophil-dependent release of vascular endothelial growth factor (VEGF) to modulate endothelial permeability [14]. Intravasating cancer cells can further collaborate with macrophages which, upon direct physical contact activate RhoA GTPase, leading to the formation of actin-rich degradative protrusions termed invadopodia [15]. In endothelial cells, the generation of reactive oxygen species (ROS) causes the disruption of adherens junctions via phosphorylation of VE-cadherin and its dissociation from complexes with β-catenin [16,17], allowing cancer cells to transmigrate through endothelial cell layers in a paracellular fashion. Furthermore, physical interaction between cancer cells and endothelial cells can activate endothelial myosin light chain (MLC) kinase which then phosphorylates myosin-II regulatory light chain followed by myosin contraction and formation of a structure called an “invasion ring”. This pore-like structure enables direct migration of cancer cells through individual endothelial cells in a transcellular manner [18].

Transforming growth factor (TGF)-β is a multifunctional cytokine that can act as a pro-metastatic by inducing EMT and local cell invasion, supporting immune evasion, stimulating angiogenesis and facilitating organotropism of seeding cancer cells to metastasis-competent distal organs [19,20]. Recent findings show a particular role of TGF-β in the transmigration of cancer cells during extravasation, i.e., the exit of disseminating tumor cells from blood vessels at distant sites. For instance, TGF-β/Smad stimulates the expression of angiopoietin-like 4 in estrogen-negative breast cancer cells which causes dissociation of cell-to-cell contacts in lung endothelial cells for successful pulmonary metastatic colonization of departing cells [21]. Another study showed that TGF-β/Smad signaling induces DOCK4—in lung adenocarcinoma cells, allowing transit of the vasculature by Rac1-mediated formation of cancer cell protrusions [22]. In pulmonary endothelial cells, TGF-β induces endothelial MLC phosphorylation associated with cytoskeletal reorganization by stress fiber formation and disintegration of barrier integrity that is either dependent or independent of the RhoA/Rho-kinase pathway [23]. A role of TGF-β was further demonstrated in vascular invasion of HCC cells using the chicken chorio-allantoic membrane assay, where HCC cells intravasate via α5β1 integrins that harbor a TGF-β/Smad-activated cytoplasmic domain of β1 integrin [24]. Yet, blood vessel invasion is still the least studied process during HCC cell dissemination due to the lack of suitable experimental models.

In this study, we established and exploited an in vitro model of hepatocellular intravasation which reflects aspects of active transmigration of invasive HCC cells through hepatic sinusoidal endothelial cells dependent on TGF-β. Using stable isotope labelling with amino acids (SILAC) of the interacting cell types and the resulting “real-time” monitoring of gene expression during transmigration, we identified molecular alterations in both endothelial as well as HCC cells and validated their relevance in HCC patients.

## 2. Results

### 2.1. Integrity and Polarization of Liver Endothelial Cells for Vascular Invasion

**Figure 1:**
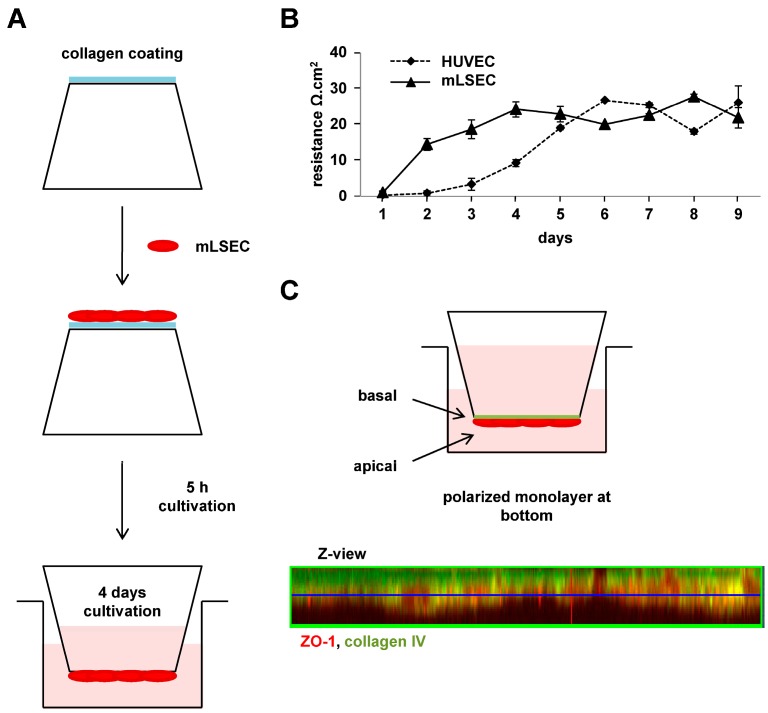
Endothelial integrity and polarity of the murine liver sinusoidal endothelial cell (mLSEC) monolayer. (**A**) Schematic drawing of plating the endothelial cells on the Transwell membrane. mLSEC cells were seeded onto the bottom of a collagen-coated Transwell membrane, allowed to attach for 5 h and subsequently cultivated upside-down for 4 days; (**B**) Transendothelial electrical resistance (TEER) analysis of mLSECs cultured upside-down onto the bottom of the Transwell membrane for up to 9 days. Human umbilical vein endothelial cells (HUVEC) cells were used as a control; (**C**) Confocal immunofluorescence analysis of ZO-1 and collagen IV showing basal and apical polarization of the mLSECs monolayer (Z-view).

Established murine liver sinusoidal endothelial cells (mLSECs) were seeded upside-down onto the bottom of collagen-coated Transwell membranes (Figure 1A). After attachment of cells for 5 h, the Transwell membranes were inserted into a 6-well plate and cultivated for 4 days. Analysis of endothelial integrity by transendothelial electrical resistance (TEER) showed increasing TEER values within 4 days of mLSEC cultivation, indicating development of endothelial cell polarity and functional tight junctions sealing the intercellular spaces in the endothelial monolayer (Figure 1B). Endothelial integrity of mLSECs was maintained between day 4 and day 9 of cultivation, similar to the TEER levels observed with established control HUVEC cells. To confirm that the polarity of the endothelial cell layer properly reflects the endothelium in situ for vascular invasion, we examined the mLSEC monolayer attached to the Transwell membrane for expression of the apical polarity marker zonula occludens (ZO)-1 and the basal polarity marker collagen IV by confocal immunofluoresecence microscopy. The Z-view of the stained endothelial monolayer showed a homogenous distribution of both ZO-1 and collagen IV (Figure 1C), indicating a basal orientation of the endothelial barrier (collagen IV) towards the top side of the Transwell membrane and an apical surface (ZO-1) towards the bottom, the latter representing the luminal side of mLSECs. Together, the data shows the tight endothelial polarity and integrity of mLSECs after 4 days of cultivation, which is suitable to study intravasation.

### 2.2. Transendothelial Migration Depends on TGF-β

**Figure 2:**
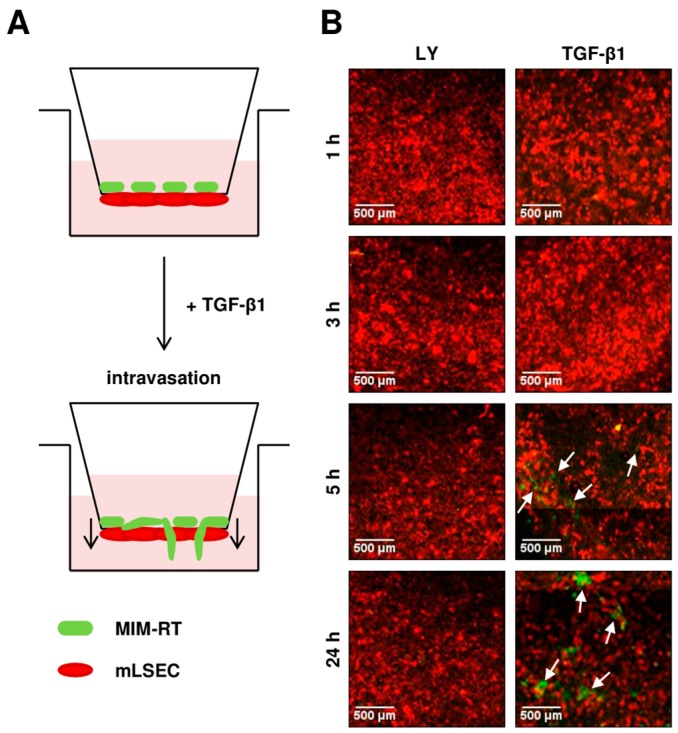
TGF-β dependent transendothelial migration. (**A**) Schematic drawing of the transendothelial migration assay. (**B**) Tile scan of transmigration kinetics. Migration of GFP-expressing MIM-RT cells across the RFP-expressing mLSECs-R monolayer, either in the presence of the TGF-β inhibitor LY2109761 (10 µM; LY) or after stimulation with 2.5 ng/mL TGF-β1 for 24 h. White arrows indicate transmigrated GFP-positive MIM-RT hepatocytes.

To study molecular changes during transendothelial migration, we plated GFP-expressing, EMT-transformed MIM-RT hepatocytes onto the top side of the Transwell membrane close to the basal domain of RFP-expressing endothelial cells, which we termed mLSEC-R (Figure 2A). In this experimental setting, the malignant MIM-RT hepatocytes require an active cell motion through the 8 µm pore size of the Transwell membrane and the endothelial mLSEC-R layer. In the next step, we determined the kinetic of transmigration in the presence and absence of TGF-β signalling. Notably, MIM-RT hepatocytes that have undergone EMT by the synergy of oncogenic H-Ras and TGF-β1 exhibit an autocrine TGF-β1 signalling loop [25]. Therefore, MIM-RT cells were either pre-treated with the TGF-β inhibitor LY2109761 for 24 h to abolish the autocrine TGF-β signalling [25] or were stimulated with 2.5 ng/mL TGF-β1 to persistently activate TGF-β/Smad signalling [26]. It has been experimentally determined that 2.5 ng/mL TGF-β1 is the lowest cytokine concentration to initiate transmigration of cells (data not shown). Interestingly, we observed transmigrated cells after 5 h of co-cultivation, whereas no transmigrating cells were detected when using MIM-RT cells treated with LY2109761 (Figure 2B). This data suggests a major role of TGF-β in the intravasation of EMT-transformed MIM-RT cells across the mLSEC endothelial barrier.

### 2.3. Molecular Alterations during Transmigration

Next, we aimed at detecting molecular changes during transmigration of MIM-RT cells through the mLSEC layer. For a quantitative analysis of protein expression, we compared mono-cultured, TGF-β-treated MIM-RT hepatocytes or mLSECs with co-cultures of MIM-RT cells and mLSECs in the same experimental setup. In order to discriminate between cell populations and to quantify protein expression of MIM-RT cells and mLSECs independently, we used four different stable isotopic labels for “real-time” analysis of protein expression by mass spectrometry as shown in Figure S1A–C.

**Figure 3:**
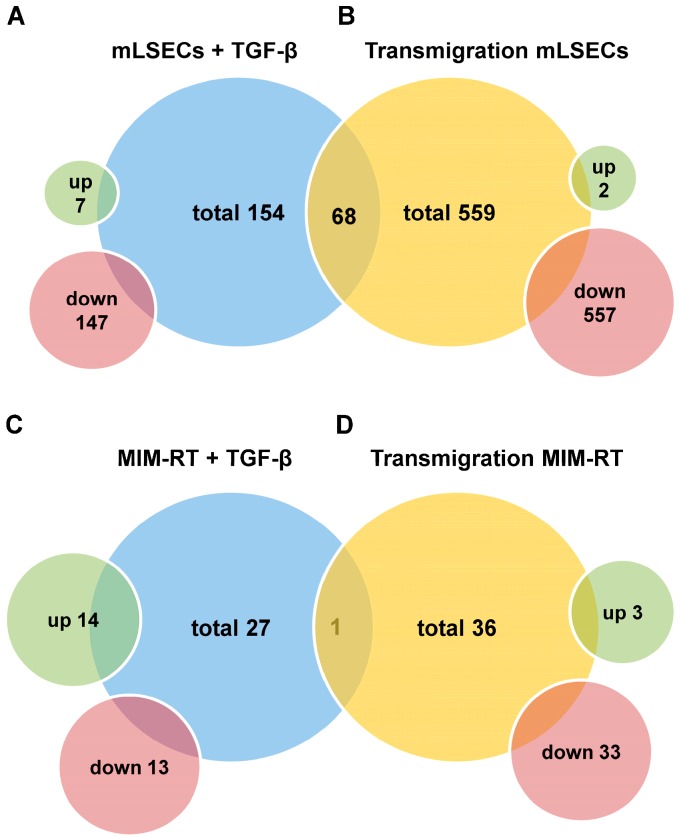
Analysis of protein expression in stable isotope labelling with amino acids (SILAC)-labeled individual and transmigrating cells by mass spectrometry. (**A**) Total number of proteins whose expression was changed in mLSECs treated with 2.5 ng/mL TGF-β1 versus those treated with 10 µM LY2109761; (**B**) Total number of proteins whose expression was altered in mLSECs during transendothelial migration; (**C**) Total number of proteins whose expression was changed in MIM-RT cells treated with 2.5 ng/mL TGF-β1 versus those treated with 10 µM LY2109761; (**D**) Total number of proteins whose expression was changed in MIM-RT cells during transendothelial migration.

To examine whether transmigration is exclusively caused by TGF-β or if there is an additional effect of cell–cell contacts in co-culture, we investigated MIM-RT cells and mLSECs in mono-culture. We detected 154 proteins with an altered expression in mLSECs (7 up- and 147 downregulated; Figure 3A, Table S1) and 27 proteins in MIM-RT cells (14 up- and 13 downregulated; Figure 3C, Table S2). Mass spectrometry analysis of mLSECs during transendothelial migration revealed 559 differentially expressed proteins (Figure 3B, Table S3). From these, 557 proteins were down- and 2 upregulated. The comparison of mLSECs treated with TGF-β1 and mLSECs during intravasation showed 68 overlapping proteins (1 variously expressed between samples, i.e., downregulated in transendothelial migraton and upregulated in mono-cultured endothelial cells; 67 downregulated). Similarly, we analyzed changes of expression in MIM-RT cells during 5 h of transmigration and detected a total number of 36 regulated proteins, 3 of them up- and 33 downregulated (Figure 3D, Table S4). The comparison between MIM-RT cells treated with TGF-β1 and MIM-RT cells during transmigration revealed 1 overlapping protein being downregulated. From this data we concluded that both EMT-transformed MIM-RT cells, as well as endothelial mLSECs undergo significant expression changes during transendothelial migration. These changes differ between transmigration and treatment of the individual cell populations with TGF-β, suggesting a significant cell–cell contact-mediated modulation of protein expression.

### 2.4. Translating Experimental Data to HCC Patients

**Figure 4:**
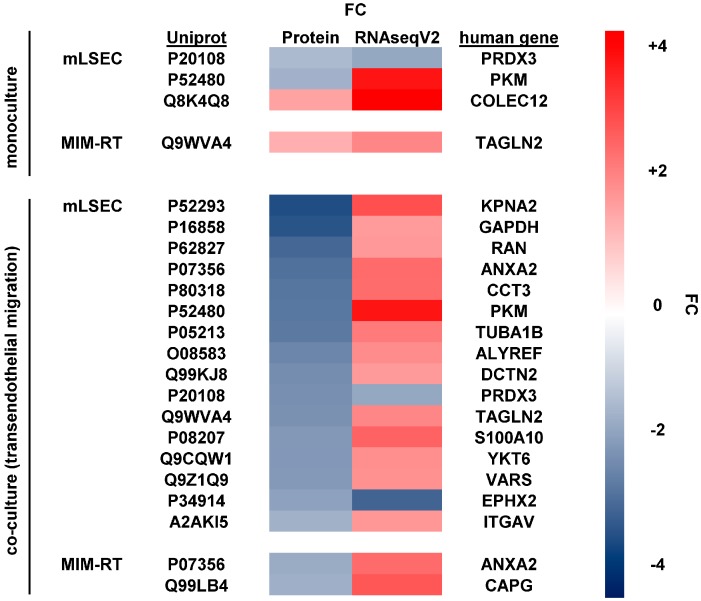
Molecular changes at the protein level in mLSECs and MIM-RT cells, as determined by mass spectrometry compared to mRNA levels of hepatocellular carcinoma (HCC) patients. The dataset RNAseqV2 from the TCGA, showing significant changes in vessel invasion and survival, was used for comparison. Displayed are targets with significant influence on the overall survival of HCC patients. Upper panel, mono-cultures of mLSECs or MIM-RT cells. Lower panel, mLSECs or MIM-RT cells analyzed during transmigration. FC, fold change.

**Figure 5:**
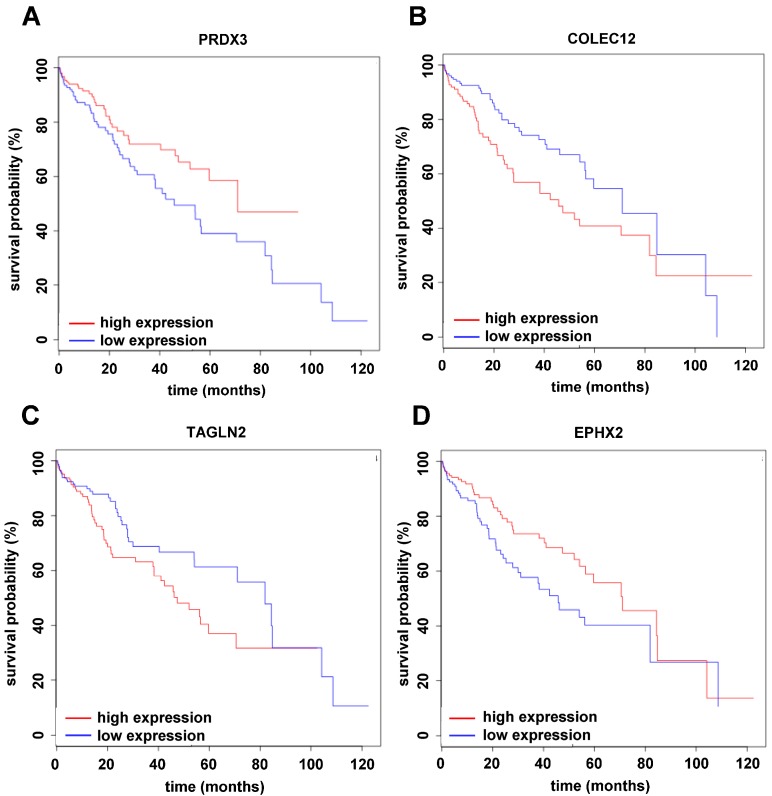
Survival analysis of (**A**) peroxiredoxin-3 (PRDX3); (**B**) collectin 12 (COLEC12); (**C**) transgelin-2 (TAGLN2) and (**D**) epoxide hydrolase (EPHX2) using the dataset RNASeq V2 from the TCGA. All RNA species showed significant differences between experimental groups; * *p* < 0.05.

In the next step, we aimed at matching our findings from cellular models with HCC patient data. Therefore, we compared the differentially expressed proteins and their corresponding genes with the RNASeqV2 dataset from the TCGA using median-based cohort sorting including 410 patient samples from 360 HCC plus 50 normal tissues followed by survival analysis. Mono-cultured mLSECs treated with TGF-β1 displayed differential expression of 3 genes with a significant influence on patient survival (Figure 4). Interestingly, peroxiredoxin-3 (PRDX3) and collectin 12 (COLEC12) displayed down- and upregulation at both the protein and RNA level in human HCC (Figure 4 and Figure 5A,B). In mono-cultured MIM-RT cells treated with TGF-β1, we detected the concomitantly increased expression of transgelin-2 (TAGLN2; Figure 4 and Figure 5C). Transmigration of MIM-RT cells through mLSECs revealed 16 changes in endothelial gene expression which are obviously relevant for patient survival. Yet, only PRDX3 and epoxide hydrolase (EPHX2) exhibited coincidence in the downregulation of protein levels and RNA levels during transmigration (Figure 4 and Figure 5D). Notably, 2 genes were found differentially expressed in EMT-transformed hepatocytes during transmigration across mLSECs, however, the regulation was shown in an opposite fashion. Together, we identified common targets when comparing our model of endothelial invasion and HCC patients with advanced stages including vessel invasion, most notably among them, PRDX3.

## 3. Discussion

Here we established and examined a homotypic model of hepato-specific transendothelial migration using EMT-transformed hepatocytes (MIM-RT) and liver sinusoidal endothelial cells (mLSECs). The characterization of both cell types has been recently reported [25,27]. In this model, (i) endothelial cells at the bottom of a Transwell membrane show polarization towards invading malignant hepatocytes mimicking an intravasation-like process during HCC metastasis [15] (Figure 1A); (ii) hepatocytes move across a tightly polarized endothelial monolayer that is separated from invasive hepatocytes by a semi-permeable membrane allowing the study of the “active” process of transmigration; (iii) alterations in protein expression can be “real-time” detected after labelling of both cell types with SILAC and subsequent analysis by mass spectrometry (Figure S1); and (iv) experimental data is translated to the HCC patient situation using a TCGA database.

MIM-RT cells represent highly invasive, metastatic hepatocytes which are induced to EMT by TGF-β1 and maintained with this phenotype by an autocrine TGF-β1 signalling loop that must be supported over time by the addition of exogenous TGF-β1 [25]. These cells escape from tumor-suppressive actions by de-differentiation through dissociation of epithelial cell–cell contacts [25]. They are able to form pulmonary metastatic colonies after orthotopic liver transplantation by showing intravasation, survival in the blood stream and extravasation (data not shown). For endothelial cells, TGF-β is simultaneously pro-apoptotic and pro-angiogenic [28], which has been explained via a transient apoptotic state mediated by VEGF/VEGF-R2 [29]. This transient apoptotic state occurs within 6–9 h upon TGF-β treatment, when 20% of cells evince apoptotic hallmarks. Notably, our analysis revealed that MIM-RT cells efficiently transmigrate through the mLSECs barrier showing functional tight junctions within 5 h upon TGF-β1 treatment (Figure 2), which is earlier than the suggested transient apoptotic state. In addition, no migration has been observed in MIM-RT cells blocked in TGF-β signalling, suggesting that active TGF-β/Smad signalling is essentially required for transmigration in this experimental model.

Our experimental setting using SILAC and mass spectrometry analysis allows us to distinguish between the protein expression in invasive hepatocytes and sinusoidal endothelial cells particularly during transmigration rather than after completion of the process. Endothelial cells treated with TGF-β1 on its own showed a significantly changed expression of 154 proteins (7 up- and 147 downregulated; Figure 3 and Table S1), while mLSECs exhibited increased alterations in protein expression during transmigration showing 559 differentially expressed proteins (2 up- and 557 downregulated; Figure 3 and Table S3). The comparison of both mLSEC expression profiles revealed 68 proteins being commonly regulated in both groups. This distinct set of regulated proteins in mLSECs is considered as a result of either a unique cellular program orchestrated by TGF-β or direct cell–cell contacts with malignant hepatocytes. Expression changes in MIM-RT cells upon TGF-β1 treatment revealed 14 up- and 13 downregulated proteins and a similar number of regulated proteins were detected in MIM-RT during transmigration (3 up- and 33 downregulated; Figure 3, Tables S2 and S4). From the number of regulated proteins in mLSECs during transmigration, we conclude that endothelial cells might be actively involved in intravasation, thus challenging the role of the endothelium as a passive barrier. Therapies based on targeting the endothelium or combined therapies could be an efficient way to treat HCC patients.

The Cancer Genome Atlas (TCGA) provides valuable RNA expression data from diseased patients which assists scientists to identify novel molecular targets and cancer biomarkers [30]. Here, we correlated the changes in protein expression obtained from our experimental model with RNA data of HCC patients and asked for those genes with a significant impact on HCC patient’s overall survival [31]. We could identify 16 genes during transmigration of mLSECs with a significant impact on patient survival. Most notably, peroxiredoxin (PRDX3; Uniprot: P20108) and epoxide hydrolase (EPHX2; Uniprot: P34914) were found to be downregulated at both protein and mRNA levels (Figure 4). In accordance with this decrease, low expression of PRDX3 or EPHX2 exhibited a strongly reduced overall survival of HCC patients (Figure 5A,D). In addition, PRDX3 but not EPHX2 was found to be regulated in the same fashion upon TGF-β treatment of mono-cultured mLSECs. Prdx3 belongs to the family of peroxiredoxin proteins which act as peroxidases by the use of electrons provided by thioredoxin [32]. In contrast to our findings and those in the TCGA database, Prdx3 was shown to be overexpressed in 39–94% of HCC cases [33,34]. A recent study further suggests that Prdx3 is an indispensable scavenger of ROS that protects HCC cells against ROS-induced damage and subsequent apoptosis, allowing favourable conditions for cancer cell proliferation and chemoresistance [35,36]. In this scenario, it is conceivable that decreased levels of PRDX3 maintains high levels of ROS that are essentially involved in the dissociation of endothelial cells by inducing phosphorylation of VE-cadherin [16,17]. Another study showed that CUL4B transgenic mice exhibit enhanced diethylnitrosamine (DEN)-induced hepatocarcinogenesis which is mediated by decreased levels of Prdx3 and increased oxidative liver damage upon DEN treatment [37]. In addition, a recent report suggests that PRDX3 is associated with tumor suppressor functions in pancreatic adenocarcinoma as its strong expression correlates with smaller tumor size, reduced invasion and negative nodal status [38]. With respect to Ephx2, which catalyses the hydrolysis of epoxide of xenobiotics to diols, its overabundance has been reported to be a marker of HCC [39]. Yet understanding the mechanistic role of Ephx2 in HCC progression is a matter for further investigation.

Notably, a common upregulation of transgelin-2 (TAGLN2; Uniprot: Q9WVA) and collectin 12 (COLEC12; Uniprot: Q8K4Q8) at both protein and mRNA levels was observed in mono-cultured MIM-RT and mLSECs cells. In addition, alterations in Tagln2 levels were displayed in transmigrating mLSECs as well, however, Tagln2 showed an opposite regulation, i.e., downregulated protein and upregulated RNA expression (Figure 4). Further, the actin-binding protein Tagln2 was suggested as a diagnostic biomarker of HCC as it is overexpressed in 69% of patients and was described to be a target of TGF-β/Smad4 in colon cancer cells [40,41]. Little is known about COLEC12 in liver pathophysiology. Yet, COLEC12 is part of the innate immune system and highly expressed in umbilical cord vascular endothelial cells. It works as a pattern recognition molecule that can act as a transmembrane receptor or may lead to opsonophagocytosis in its soluble form [42].Significantly, COLEC12 binds sialyl Lewis X which interacts with E-selectin during extravasation of leukocytes and cancer cells [43]. Patients with colorectal cancer show activation of the lectin-complement pathway that works similar to collectins, and concomitantly exhibit increased levels of mannose-binding lectin [44]. Further studies on COLEC12 are needed to understand its role in hepatocarcinogenesis.

Among 16 targets regulated in mLSECs and 2 targets regulated in MIM-RT cells during intravasation (Figure 4), we could observe an opposite trend where proteins were downregulated and mRNA level upregulated. It should be noted that TCGA uses a comparison between samples from tumor tissue versus non-tumor tissue from healthy individuals and HCC patients. As opposed to this, we compared HCC cells treated with a TGF-β inhibitor as a reference and migrating HCC cells stimulated with TGF-β as the subject of interest. The fact that the detected expression changes at the protein level in intravasating HCC cells do not correspond to mRNA expression obtained from primary HCC cells could also be explained by similar trends observed in circulating tumor cells, which vary in their expression profiles from residing cancer cells [45].

Recent studies emphasize differential dynamics of mRNA and protein expression, and suggest that regulations on the protein level might be more important for phenotypic adaptation than transcriptomic changes [46]. In addition, a substantial limitation of our study must be taken into consideration. Phosphorylation and dephosphorylation of proteins represent important mechanisms in the regulation of cellular response. However, the detection and quantification of phosphorylated proteins is still far from becoming routine in mass spectrometry [47].

Our results suggest a key role of TGF-β during endothelial transmigration and highlight the active contribution of endothelial cells in this process. Endothelial cells undergo multiple expression changes which call their role as a passive barrier into question. Directly targeting the disintegration of the endothelium, either alone or as part of a combined therapy, could have a valuable therapeutic potential in preventing intra- and extrahepatic metastasis of HCC cells. Moreover, intravasating cancer cells undergo expression changes which vary from individual cell migration driven by TGF-β and from solid tumor tissue samples, thus representing a unique cellular program. The validation of the phenotypical impact of these identified proteins and their involvement in cellular and molecular mechanisms requires further experimental evaluation by gain- and loss-of-function studies. Discrepancies between TCGA and proteomics data indicate that a combined-omics approaches at all stages of the metastatic cascade provide deeper insights into the molecular mechanisms of cancer development.

## 4. Materials and Methods

### 4.1. Cell Culture

The murine p19^ARF^-deficient, EMT-transformed MIM-RT hepatocytes expressing green fluorescent protein (GFP) were cultivated in RPMI 1640 plus 10% fetal calf serum (FCS) and continuously supplied with 1 ng/mL TGF-β1 (Peprotech, Rocky Hill, NJ, USA) as described previously [25]. The murine p19^ARF^-deficient liver sinusoidal endothelial cells, termed mLSECs, were propagated on collagen-coated (Collagen Type I-Rat Tail, BD Biosciences, San Jose, CA, USA, Cat.#354236) petri dishes in Dulbecco’s Modified Eagle’s Medium (DMEM) containing 100 µg/mL Endothelial Cell Growth Supplement (ECGS; Biomedical Technologies, Stoughton, MA, USA, Cat.#BT203), 0.2 µg/mL hydrocortisone (Alfa/Aesar, Karlsruhe, Germany, Cat.#A16292), and 50 µg/mL heparin (AppliChem, Darmstadt, Germany, #3U009511) as outlined recently [27]. For fluorescent labelling, mLSECs cells were lentivirally transmitted with a vector harbouring red fluorescent protein (RFP), resulting in mLSECs-R cells. Human umbilical vein endothelial cells (HUVECs) were grown in Endothelial Cell Medium (ECM; ScienCell, Carlsbad, CA, USA, Cat.#1001) following the manufacturer’s instruction. All cells were grown at 37 °C and 5% CO_2_, and were routinely screened for the absence of mycoplasma.

### 4.2. Cultivation of Cells on Transwell Membrane

Twenty-four mm Transwell permeable supports (Corning, New York, NY, USA) were placed upside-down, coated with collagen (Collagen Type I-Rat Tail, BD Biosciences, Cat.#354236) on the bottom and allowed to air-dry under sterile conditions. 1 mL of mLSEC suspension containing 5 × 10^5^ cells was seeded upside-down onto the bottom of the Transwell membrane and placed into the humidified incubator for 5 h to allow attachment of the cells. After attachment, the Transwell membranes were inserted into 6-well plates containing endothelial growth medium and cells were allowed to proliferate for 4 days. Subsequently, the endothelial medium was changed and 5 × 10^5^ MIM-RT cells were seeded on top of the Transwells in medium containing 2.5 ng/mL TGF-β1 (Peprotech).

### 4.3. Transmigration Kinetics

MIM-RT cells stably expressing GFP were added on top of the Transwell membranes (Corning, New York, NY, USA) that was covered with a monolayer of RFP-expressing mLSECs (mLSECs-R) on the bottom of the Transwell. The transmigration was performed in the presence of 2.5 ng/mL TGF-β1 for 1, 2, 3, 4, 5 and 24 h. The Transwell filter inserts were subsequently fixed with 4% phosphate-buffered formalin and analyzed by confocal fluorescence microscopy (Zeiss, Oberkochen, Germany) using the tile scan function to obtain high resolution large field images.

### 4.4. Transendothelial Electrical Resistance

The transendothelial electrical resistance (TEER) of mLSECs monolayers at the bottom of the Transwell membrane was analyzed as described previously [48]. The growth medium was changed every second day and the resistance was determined daily with a volt-ohm meter. All TEER values were normalized to background values, i.e., Transwell filter in growth medium only.

### 4.5. Confocal Immunofluoresecence Microscopy

Cells were seeded on collagen-coated Transwell membranes (Corning) and fixed with 4% formaldehyde. After permeabilization with 0.25% Triton-X 100 and blocking, cells were stained with primary antibody against ZO-1 (Zymed Laboratories, South San Francisco, CA, USA) and collagen IV (Santa Cruz, Dallas, TX, USA) at a concentration of 1:75 and 1:100, respectively, and further incubated with secondary antibody (1:200). Images were obtained by confocal immunofluorescence microscopy (Zeiss).

### 4.6. Stable Isotope Labelling with Amino Acids (SILAC)

mLSECs and MIM-RT cells were cultivated in corresponding SILAC-media following the manufacturer’s instructions (Thermo Fisher Scientific, Waltham, MA, USA). Briefly, MIM-RT cells were cultivated in SILAC-RPMI medium containing l-lysine–2HCl ^13^C_6_ (MIM-RT Heavy) and l-lysine–2HCl ^13^C_6_^15^N_2_ (MIM-RT Super-Heavy), respectively. mLSECs were cultivated in SILAC-DMEM medium containing l-arginine–HCl ^13^C_6_^15^N_4_ (mLSECs Heavy) and SILAC-DMEM without heavy isotopes (mLSECs Light).

### 4.7. Electrophoresis and In-Gel Digestion

The cell lysate samples corresponding to the same group (3 samples TGF-β1-treated and untreated each, 5 h incubation time) were pooled so that each sample in the group contributed with the same amount of total protein. The group samples were heated to 95 °C for 5 min and cooled on ice prior to loading onto NuPAGE 4–12% Bis-Tris gels (Thermo Fisher Scientific). SDS polyacrylamide gelelectrophoresis (SDS-PAGE) was performed according to the manufacturer’s specifications. Proteins were fixed within the polyacrylamide matrix by incubating the entire gel in 5% acetic acid in 1:1 water:methanol for 30 min. After Coomassie staining, the gel slab was rinsed with water and each lane was excised and cut into small pieces. Subsequently, the proteins were in-gel destained (100 mM ammonium bicarbonate/acetonitrile 1:1), reduced (10 mM DTT), alkylated (50 mm iodoacetamide) and finally trypsin-digested overnight at 37 °C. The generated peptides were collected from the gel pieces which were further subjected to a peptide extraction step with an acidic (1.5% formic acid) acetonitrile (66%) solution. Both peptides containing samples were combined and dried in a vacuum centrifuge.

### 4.8. Mass Spectrometry

The dried peptides were re-dissolved in 0.1% trifluoroacetic acid and loaded on a C18 precolumn (Acclaim; Dionex, Sunnyvale, CA, USA) using an RSLCnano HPLC system (Dionex). Peptides were then eluted with an aqueous-organic gradient, resolved on a C18 column (Acclaim; Dionex, Sunnyvale, CA, USA) with a flow rate of 300 nL/min and electro sprayed into an LTQ Orbitrap XL mass spectrometer (Thermo Scientific). A Triversa Automate (Advion Biosciences, Ithaca, NY, USA) was used as ion source. Each scan cycle consisted of one FTMS full scan and up to seven ITMS dependent MS/MS scans of the seven most intense ions. Dynamic exclusion (30 s), mass width (10 ppm) and monoisotopic precursor selection were enabled. All analyses were performed in positive ion mode. Extracted MS/MS spectra were searched against the Uniprot/Swissprot database using the PEAKS search engine (Bioinformatics Solutions Inc., Waterloo, ON, Canada) accepting common variable modifications and one missed tryptic cleavage. Peptide tolerance was ±10 ppm and MS/MS tolerance was ±0.5 Da. All protein identification experiments were carried out using the corresponding decoy database and a false discovery rate (FDR) of 1%. The SILAC precursor ion quantification of the proteins was performed with the SILAC quantification tool of the PEAKS Studio Software (Bioinformatics Solutions Inc) using a mass error tolerance of ±0.1 Da and a retention time shift of ±0.5 min.

### 4.9. Comparison of Differentially Expressed Genes with TCGA Data

Orthologous genes between mice and humans were mapped using the Ensembl genome browser in order to obtain the human Ensembl gene IDs and human gene symbols for comparison with TCGA (The Cancer Genome Atlas) gene expression data. The RNASeq version2 data for HCC were downloaded from the TCGA Data Portal (https://tcga-data.nci.nih.gov/tcga/, July 2015 release). For RNASeqV2, 410 samples with clinical patient information were available (50 normal solid tissue and 360 primary solid tumor samples). Raw counts from gene level data were used for the analysis of differentially expressed genes using the Bioconductors “edgeR” package. Gene counts were normalized using edgeR’s TMM (trimmed mean of *M* values). Differential gene expression between tumor and normal samples of RNASeqV2 sample data, respectively, was assessed using edgeR’s exact test.

### 4.10. Survival Analysis Using Differentially Expressed Genes

The survival analysis was performed in R using the “survival” package for those proteins which were differentially expressed in mouse MS data as well as in the TCGA RNAseq data set using a cut-off for differentially expressed genes with a minimum of 1.5-fold change in same direction, and an adjusted *p*-value < 0.05. Therefore, RPKM values were calculated from TMM-normalized counts using edgeR’s RPKM function. In order to split the patients into two groups with different survival probabilities exhibiting higher or lower gene expression, two different approaches were used: (1) For each gene, the patients were split into high- or low-expressing groups according to whether the expression of the candidate gene was greater than the median expression of the candidate gene; (2) For each gene, the patients were split into high- or low-expressing groups using maximally selected rank statistics as implemented in the maxstat R package. The statistical significance of differences in overall survival between the two groups was calculated by the log-rank test, and survival curves were plotted using the Kaplan–Meier method.

### 4.11. Statistics

The data are expressed as mean ± standard deviation. The statistical significance of differences was evaluated using a paired, non-parametric Student’s *t*-test. Significant differences between experimental groups were * *p* < 0.05.

## Supplementary Materials

Supplementary materials can be found at www.mdpi.com/1422-0067/18/10/2119/s1.

## Abbreviations

BCLC	Barcelona Clinic Liver Cancer
EMT	Epithelial to mesenchymal transition
COLEC12	Collectin 12
EPHX2	Epoxide hydrolase
GFP	Green fluorescent protein
HCC	Hepatocellular carcinoma
HUVEC	Human umbilical vein endothelial cell
MLC	Myosin light chain
mLSEC	Murine liver sinusoidal endothelial cell
PRDX3	Peroxiredoxin-3
RFP	Red fluorescent protein
ROS	Reactive oxygen species
SILAC	Stable isotope labelling with amino acids
TCGA	The cancer genome atlas
TEER	Transendothelial electrical resistance
TGF	Transforming growth factor
TAGLN2	Transgelin-2
VEGF	Vascular endothelial growth factor
ZO-1	Zonula occludens 1
