# Supplementary material for: Transforming Growth Factor-β Drives the Transendothelial Migration of Hepatocellular Carcinoma Cells
Source: Int J Mol Sci. 2017 Oct 10;18(10):2119. doi: 10.3390/ijms18102119 (PMC5666801; doi:10.3390/ijms18102119)
Supplement: Supplementary file 1 [file ijms-18-02119-s001.pdf]

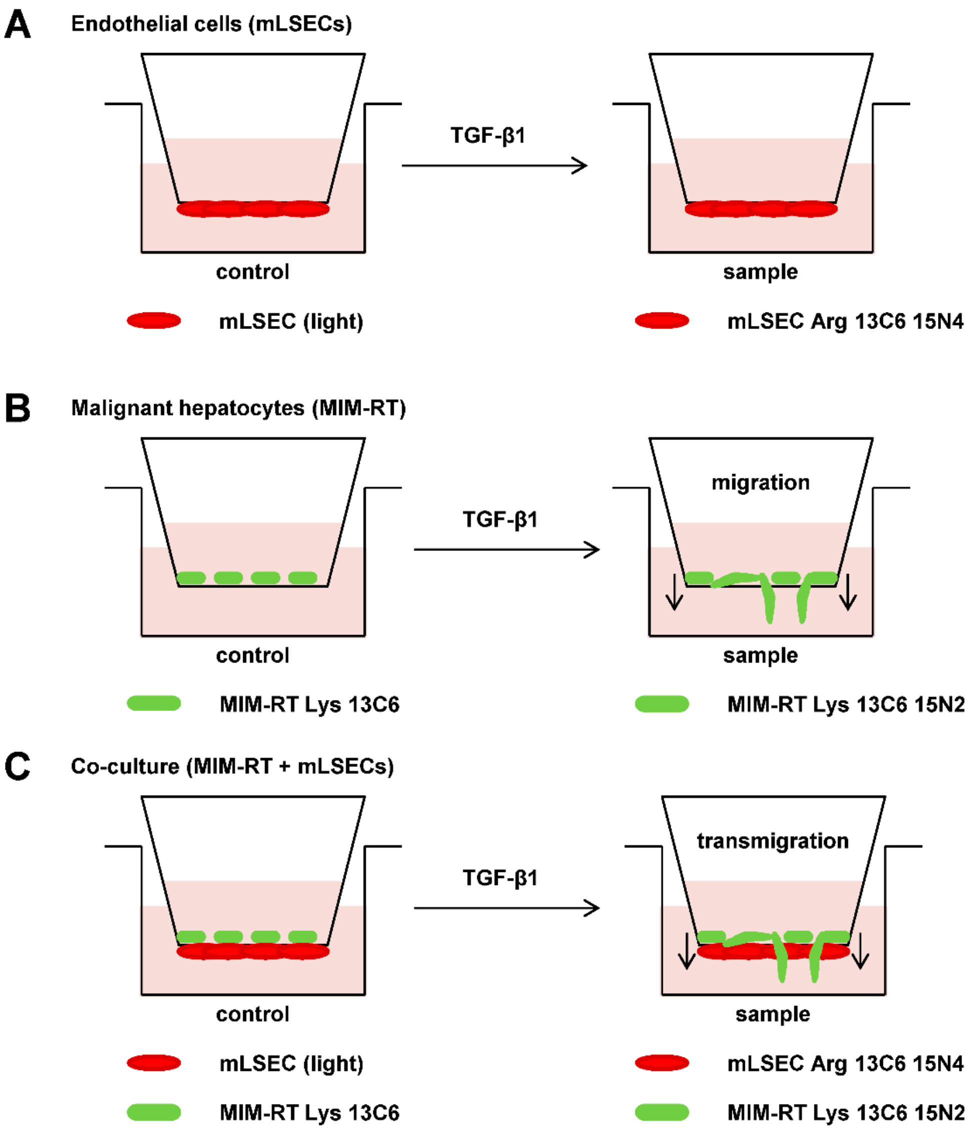

Figure S1. Experimental setup of SILAC-based mass spectrometry analysis. (**A**) Unlabeled endothelial cells serve as a control, whereas TGF-β1 treated cells were labeled with arginine 13C6 15N4. (**B**) Migration of MIM-RT cells labeled with lysine 13C6 as a control and lysine 13C6 15N2, respectively. (**C**) Transmigration of MIM-RT cells through endothelial mLSEC cells using four different SILAC labels. All SILAC-labeled cells were analyzed by mass spectrometry.

Table S1. Differentially expressed proteins in mLSEC treated with TGF-β1.

Accession	Description	Expression Ratio Sample/Control
Q9JIG8	PRA1 family protein 2	1.84
Q60854	Serpin B6	1.72
Q9DBR7-2	Isoform 2 of Protein phosphatase 1 regulatory subunit 12A	1.52
Q9D1J3	SAP domain-containing ribonucleoprotein	1.50
Q8K4Q8	Collectin-12	1.48
P24549	Retinal dehydrogenase 1	1.45
P62320	Small nuclear ribonucleoprotein Sm D3	1.39
Q9D0I9	Arginine--tRNA ligase_ cytoplasmic	0.29
Q9WVJ3-2	Isoform 2 of Carboxypeptidase Q	0.30
P61979	Heterogeneous nuclear ribonucleoprotein K	0.34
Q9CQ60	6-phosphogluconolactonase	0.48
Q9CQJ8	NADH dehydrogenase [ubiquinone] 1 beta subcomplex subunit 9	0.49
Q99020	Heterogeneous nuclear ribonucleoprotein A/B	0.51
Q60931	Voltage-dependent anion-selective channel protein 3	0.52
J3KMM5	Calcium-transporting ATPase	0.53
Q8K411-3	Isoform 3 of Presequence protease_ mitochondrial	0.54
A0A0B4J1M9	Microtubule-associated protein 4	0.55
P37040	NADPH--cytochrome P450 reductase	0.55
P01027	Complement C3	0.56
E9QKZ2	Importin-9	0.56
Q99M71	Mammalian ependymin-related protein 1	0.56
Q9D7B7	Probable glutathione peroxidase 8	0.56
Q64727	Vinculin	0.56
A2A9X5	5'(3')-deoxyribonucleotidase_ cytosolic type	0.57
Q61599	Rho GDP-dissociation inhibitor 2	0.57
Q9Z2C6	Uroplakin-1b	0.57
Q8BIJ6	Isoleucine--tRNA ligase_ mitochondrial	0.58
P08003	Protein disulfide-isomerase A4	0.58
O89023	Tripeptidyl-peptidase 1	0.58
P61222	ATP-binding cassette sub-family E member 1	0.61
D3YW48	Calpain small subunit 1 (Fragment)	0.61
O09131	Glutathione S-transferase omega-1	0.61
Q9DCN2-2	Isoform 2 of NADH-cytochrome b5 reductase 3	0.61
P29758	Ornithine aminotransferase_ mitochondrial	0.61
Q8BXC0	Prostacyclin synthase	0.61
Q5NCU4	SPARC	0.61
Q9CXW3	Calcyclin-binding protein	0.62
B7ZNJ1	Fibronectin	0.63
Q99KI0	Aconitate hydratase_ mitochondrial	0.64
Q99MN1	Lysine--tRNA ligase	0.64
Q7TMF3	NADH dehydrogenase [ubiquinone] 1 alpha subcomplex subunit 12	0.64
Q99K94	Signal transducer and activator of transcription	0.64
P56380	Bis(5'-nucleosyl)-tetraphosphatase [asymmetrical]	0.65
B1AWE0	Clathrin light chain A	0.65
P09528	Ferritin heavy chain	0.65

Q504P4	Heat shock cognate 71 kDa protein	0.65
Q8BMS1	Trifunctional enzyme subunit alpha_ mitochondrial	0.65
Q91ZJ5	UTP--glucose-1-phosphate uridylyltransferase	0.65
Q9DBX5	Cytosolic phospholipase A2	0.66
P45952	Medium-chain specific acyl-CoA dehydrogenase_ mitochondrial	0.66
Q9CQZ5	NADH dehydrogenase [ubiquinone] 1 alpha subcomplex subunit 6	0.66
Q5SQB0	Nucleophosmin	0.66
Q3TXS7	26S proteasome non-ATPase regulatory subunit 1	0.67
P62855	40S ribosomal protein S26	0.67
P47738	Aldehyde dehydrogenase_ mitochondrial	0.67
Q64133	Amine oxidase [flavin-containing] A	0.67
P60766	Cell division control protein 42 homolog	0.67
O88544	COP9 signalosome complex subunit 4	0.67
P62897	Cytochrome c_ somatic	0.67
P70372	ELAV-like protein 1	0.67
P70699	Lysosomal alpha-glucosidase	0.67
P07742	Ribonucleoside-diphosphate reductase large subunit	0.67
Q9R0P3	S-formylglutathione hydrolase	0.67
Q921L3	Transmembrane and coiled-coil domain-containing protein 1	0.67
Q9WUL7	ADP-ribosylation factor-like protein 3	0.68
Q922B2	Aspartate--tRNA ligase_ cytoplasmic	0.68
Q9Z2W0	Aspartyl aminopeptidase	0.68
Q922D8	C-1-tetrahydrofolate synthase_ cytoplasmic	0.68
O08749	Dihydrolipoyl dehydrogenase_ mitochondrial	0.68
Q9ER72-2	Isoform 2 of Cysteine--tRNA ligase_ cytoplasmic	0.68
Q9EQ20	Methylmalonate-semialdehyde dehydrogenase [acylating]_ mitochondrial	0.68
O55131	Septin-7	0.68
P28653	Biglycan	0.69
Q01149	Collagen alpha-2(I) chain	0.69
Q3UJB0	Protein Sf3b2	0.69
A0A087WQE6	Transcription elongation factor B polypeptide 1 (Fragment)	0.69
Q99KK7	Dipeptidyl peptidase 3	0.70
E9QN08	Elongation factor 1-delta (Fragment)	0.70
Q9EQK5	Major vault protein	0.70
Q9DC70	NADH dehydrogenase [ubiquinone] iron-sulfur protein 7_ mitochondrial	0.70
Q3THS6	S-adenosylmethionine synthase isoform type-2	0.70
Q64737	Trifunctional purine biosynthetic protein adenosine-3	0.70
O88342	WD repeat-containing protein 1	0.70
Q9CPY7	Cytosol aminopeptidase	0.71
Q99K01-3	Isoform 3 of Pyridoxal-dependent decarboxylase domain-containing protein 1	0.71
P52480	Pyruvate kinase PKM	0.71
Q9CQA3	Succinate dehydrogenase [ubiquinone] iron-sulfur subunit_ mitochondrial	0.71
H3BJY1	Synaptogyrin-2	0.71
Q80YF6	Uroplakin-3b	0.71

Q63829	COMM domain-containing protein 3	0.72
Q9DBF1-2	Isoform 2 of Alpha-aminoadipic semialdehyde dehydrogenase	0.72
P11983-2	Isoform 2 of T-complex protein 1 subunit alpha	0.72
P97807-2	Isoform Cytoplasmic of Fumarate hydratase_ mitochondrial	0.72
A2AIW9	Mitochondrial-processing peptidase subunit alpha	0.72
P22437	Prostaglandin G/H synthase 1	0.72
Q62465	Synaptic vesicle membrane protein VAT-1 homolog	0.72
H3BKL5	3-ketoacyl-CoA thiolase A_ peroxisomal	0.73
P40124	Adenylyl cyclase-associated protein 1	0.73
D3YYT0	Cadherin-2	0.73
D3Z368	Calcium/calmodulin-dependent protein kinase type 1 (Fragment)	0.73
Q8VI75	Importin-4	0.73
Q78IK4	MICOS complex subunit Mic27	0.73
D3YXC2	Mitochondrial carrier homolog 1 (Fragment)	0.73
Q9D358-2	Isoform 2 of Low molecular weight phosphotyrosine protein phosphatase	0.74
Q91VS7	Microsomal glutathione S-transferase 1	0.74
O55125	Protein NipSnap homolog 1	0.74
A2A547	Ribosomal protein L19	0.74
P57746	V-type proton ATPase subunit D	0.74
P35564	Calnexin	0.75
Q02248	Catenin beta-1	0.75
Q7JCZ1	Cytochrome c oxidase subunit 2	0.75
Q62425	Cytochrome c oxidase subunit NDUFA4	0.75
O55022	Membrane-associated progesterone receptor component 1	0.75
P35700	Peroxiredoxin-1	0.75
Q8VEM8	Phosphate carrier protein_ mitochondrial	0.75
Q543K9	Purine nucleoside phosphorylase	0.75
Q8VEE0	Ribulose-phosphate 3-epimerase	0.75
P62715	Serine/threonine-protein phosphatase 2A catalytic subunit beta isoform	0.75
P80313	T-complex protein 1 subunit eta	0.75
P12970	60S ribosomal protein L7a	0.76
O08529	Calpain-2 catalytic subunit	0.76
Q3T9X3	Dynamin-2	0.76
P10126	Elongation factor 1-alpha 1	0.76
Q8C253	Galectin	0.76
Q76MZ3	Serine/threonine-protein phosphatase 2A 65 kDa regulatory subunit A alpha isoform	0.76
A2AUM6	Signal recognition particle 14 kDa protein	0.76
Q9WV55	Vesicle-associated membrane protein-associated protein A	0.76
Q9CPQ8	ATP synthase subunit g_ mitochondrial	0.77
Q99JZ4	GTP-binding protein SAR1a	0.77
O88587-2	Isoform Soluble of Catechol O-methyltransferase	0.77
E9PZF0	Nucleoside diphosphate kinase	0.77
Q01853	Transitional endoplasmic reticulum ATPase	0.77
Q9CZ04	COP9 signalosome complex subunit 7a	0.78

Q8BKC5	Importin-5	0.78
Q99PT1	Rho GDP-dissociation inhibitor 1	0.78
F8WHP8	ATP synthase subunit f_ mitochondrial	0.79
G5E850	Cytochrome b-5_ isoform CRA_a	0.79
P16045	Galectin-1	0.79
Q9DCF9-2	Isoform 2 of Translocon-associated protein subunit gamma	0.79
Q8BJS4-3	Isoform 3 of SUN domain-containing protein 2	0.79
P99024	Tubulin beta-5 chain	0.79
Q8BYA0	Tubulin-specific chaperone D	0.79
Q9CZU6	Citrate synthase_ mitochondrial	0.80
P00493	Hypoxanthine-guanine phosphoribosyltransferase	0.80
Q9D071-3	Isoform 3 of MMS19 nucleotide excision repair protein homolog	0.80
Q9DCL9	Multifunctional protein ADE2	0.80
Q99JR1	Sideroflexin-1	0.80
P20108	Thioredoxin-dependent peroxide reductase_ mitochondrial	0.80
Q99KC8	von Willebrand factor A domain-containing protein 5A	0.80
Q9WVJ2	26S proteasome non-ATPase regulatory subunit 13	0.81
Q8VDM4	26S proteasome non-ATPase regulatory subunit 2	0.81
Q8VDD5	Myosin-9	0.81
Q9DCT2	NADH dehydrogenase [ubiquinone] iron-sulfur protein 3_ mitochondrial	0.81
Q8K310	Matrin-3	0.84

Table S2. Differentially expressed proteins in MIM-RT cells treated with TGF-β1.

Uniprot ID	Description	Expression Ratio Sample/Control
P60229	Eukaryotic translation initiation factor 3 subunit E	1.66
P62849	40S ribosomal protein S24	1.41
Q9CQQ7	ATP synthase F(0) complex subunit B1_ mitochondrial	1.38
P47754	F-actin-capping protein subunit alpha-2	1.36
Q9JJI8	60S ribosomal protein L38	1.35
Q99020	Heterogeneous nuclear ribonucleoprotein A/B	1.35
Q6ZWZ7	60S ribosomal protein L17	1.32
Q9JKB3-2	Isoform 2 of Y-box-binding protein 3	1.32
Q9WVA4	Transgelin-2	1.30
P20029	78 kDa glucose-regulated protein	1.27
P07091	Protein S100-A4	1.27
P68040	Guanine nucleotide-binding protein subunit beta-2-like 1	1.26
Q91WK2	Eukaryotic translation initiation factor 3 subunit H	1.25
P14602-2	Isoform B of Heat shock protein beta-1	1.25
P09528	Ferritin heavy chain	0.55
P11679	Keratin_ type II cytoskeletal 8	0.63
Q9CQX2	Cytochrome b5 type B	0.65
Q6PB66	Leucine-rich PPR motif-containing protein_ mitochondrial	0.66
P51660	Peroxisomal multifunctional enzyme type 2	0.68
P10649	Glutathione S-transferase Mu 1	0.71
P99029-2	Isoform Cytoplasmic+peroxisomal of Peroxiredoxin-5_ mitochondrial	0.71
Q64674	Spermidine synthase	0.73
P59325	Eukaryotic translation initiation factor 5	0.74
Q9DCD0	6-phosphogluconate dehydrogenase_ decarboxylating	0.77
E9Q616	Protein Ahnak	0.78
P50247	Adenosylhomocysteinase	0.79
P50580-2	Isoform 2 of Proliferation-associated protein 2G4	0.79

Table S3. Differentially expressed proteins during transmigration of mLSECs.

Uniprot ID	Description	Expression Ratio Sample/Control
Q02819	Nucleobindin-1	1.25
Q66GT5	Phosphatidylglycerophosphatase and protein-tyrosine phosphatase 1	1.20
Q61937	Nucleophosmin	0.22
Q9R0P3	S-formylglutathione hydrolase	0.25
Q9QYJ0	DnaJ homolog subfamily A member 2	0.26
O09131	Glutathione S-transferase omega-1	0.26
A2BE93	Protein SET (Fragment)	0.26
P14206	40S ribosomal protein SA	0.27
P08030	Adenine phosphoribosyltransferase	0.27
Q6GT24	Peroxiredoxin 6	0.28
Q9R1Q7	Proteolipid protein 2	0.28
Q9CX56	26S proteasome non-ATPase regulatory subunit 8	0.29
P05201	Aspartate aminotransferase_ cytoplasmic	0.30
P07901	Heat shock protein HSP 90-alpha	0.30
Q9ER72-2	Isoform 2 of Cysteine--tRNA ligase_ cytoplasmic	0.30
P16546-2	Isoform 2 of Spectrin alpha chain_ non-erythrocytic 1	0.30
Q9DC70	NADH dehydrogenase [ubiquinone] iron-sulfur protein 7_ mitochondrial	0.30
Q9CQR4	Acyl-coenzyme A thioesterase 13	0.31
P70168	Importin subunit beta-1	0.31
Q8BJY1	26S proteasome non-ATPase regulatory subunit 5	0.32
E9Q242	Adenylosuccinate lyase	0.32
Q9CQX2	Cytochrome b5 type B	0.32
Q80X90	Filamin-B	0.32
P52293	Importin subunit alpha-1	0.32
Q9QXS1-15	Isoform PLEC-1H of Plectin	0.32
A2AIW9	Mitochondrial-processing peptidase subunit alpha	0.32
Q60854	Serpin B6	0.32
Q8BP47	Asparagine--tRNA ligase_ cytoplasmic	0.33
Q8C166	Copine-1	0.33
E9PWE8	Dihydropyrimidinase-related protein 3	0.33
Q8BL66	Early endosome antigen 1	0.33
P60229	Eukaryotic translation initiation factor 3 subunit E	0.33
P16858	Glyceraldehyde-3-phosphate dehydrogenase	0.33
P52760	Ribonuclease UK114	0.33
Q62465	Synaptic vesicle membrane protein VAT-1 homolog	0.33
Q9D0R2	Threonine--tRNA ligase_ cytoplasmic	0.33
G3UZ34	116 kDa U5 small nuclear ribonucleoprotein component	0.34
P46664	Adenylosuccinate synthetase isozyme 2	0.34
P35564	Calnexin	0.34
O55135	Eukaryotic translation initiation factor 6	0.34
Q9CZD3	Glycine--tRNA ligase	0.34

P24547	Inosine-5'-monophosphate dehydrogenase 2	0.34
Q9Z110-2	Isoform Short of Delta-1-pyrroline-5-carboxylate synthase	0.34
A2AT02	NSFL1 cofactor p47	0.34
O54724	Polymerase I and transcript release factor	0.34
Q9R0X4	Acyl-coenzyme A thioesterase 9_ mitochondrial	0.35
Q8BGQ7	Alanine--tRNA ligase_ cytoplasmic	0.35
P11499	Heat shock protein HSP 90-beta	0.35
P48678	Prelamin-A/C	0.35
Q8VEE0	Ribulose-phosphate 3-epimerase	0.35
P68372	Tubulin beta-4B chain	0.35
P63325	40S ribosomal protein S10	0.36
Q9WUL7	ADP-ribosylation factor-like protein 3	0.36
P60843	Eukaryotic initiation factor 4A-I	0.36
Q8C854	Myelin expression factor 2	0.36
Q9CPP0	Nucleoplasmin-3	0.36
Q9JKF1	Ras GTPase-activating-like protein IQGAP1	0.36
A2A547	Ribosomal protein L19	0.36
P68369	Tubulin alpha-1A chain	0.36
E9PYI8	Ubiquitin carboxyl-terminal hydrolase	0.36
Q5RKN9	Capping protein (Actin filament) muscle Z-line_ alpha 1	0.37
P57776	Elongation factor 1-delta	0.37
P62827	GTP-binding nuclear protein Ran	0.37
P00493	Hypoxanthine-guanine phosphoribosyltransferase	0.37
Q791T5-2	Isoform 2 of Mitochondrial carrier homolog 1	0.37
Q9CYG7	Mitochondrial import receptor subunit TOM34	0.37
Q91YZ8	Polyadenylate-binding protein	0.37
P62264	40S ribosomal protein S14	0.38
P62843	40S ribosomal protein S15	0.38
Q9JM14	5'(3')-deoxyribonucleotidase_ cytosolic type	0.38
P47911	60S ribosomal protein L6	0.38
Q9EST5	Acidic leucine-rich nuclear phosphoprotein 32 family member B	0.38
Q9D898	Actin-related protein 2/3 complex subunit 5-like protein	0.38
E9QNN1	ATP-dependent RNA helicase A	0.38
Q8BMF4	Dihydrolipoyllysine-residue acetyltransferase component of pyruvate dehydrogenase complex_ mitochondrial	0.38
P23116	Eukaryotic translation initiation factor 3 subunit A	0.38
Q9DCH4	Eukaryotic translation initiation factor 3 subunit F	0.38
Q8BKC5	Importin-5	0.38
P50580-2	Isoform 2 of Proliferation-associated protein 2G4	0.38
P60335	Poly(rC)-binding protein 1	0.38
P99026	Proteasome subunit beta type-4	0.38
Q62093	Serine/arginine-rich splicing factor 2	0.38
Q64674	Spermidine synthase	0.38
P26516	26S proteasome non-ATPase regulatory subunit 7	0.39
P97351	40S ribosomal protein S3a	0.39
P47963	60S ribosomal protein L13	0.39

P19253	60S ribosomal protein L13a	0.39
Q9CR57	60S ribosomal protein L14	0.39
P12970	60S ribosomal protein L7a	0.39
P40124	Adenylyl cyclase-associated protein 1	0.39
P07356	Annexin A2	0.39
O08749	Dihydrolipoyl dehydrogenase_ mitochondrial	0.39
O70194	Eukaryotic translation initiation factor 3 subunit D	0.39
Q9D6R2	Isocitrate dehydrogenase [NAD] subunit alpha_ mitochondrial	0.39
Q61990	Poly(rC)-binding protein 2	0.39
Q9Z2U0	Proteasome subunit alpha type-7	0.39
Q9Z2I9	Succinyl-CoA ligase [ADP-forming] subunit beta_ mitochondrial	0.39
P26039	Talin-1	0.39
P80317	T-complex protein 1 subunit zeta	0.39
E9Q5I9	26S proteasome non-ATPase regulatory subunit 13	0.40
Q8VDM4	26S proteasome non-ATPase regulatory subunit 2	0.40
P62852	40S ribosomal protein S25	0.40
P62242	40S ribosomal protein S8	0.40
P35979	60S ribosomal protein L12	0.40
Q9CQM8	60S ribosomal protein L21	0.40
P59999	Actin-related protein 2/3 complex subunit 4	0.40
P48036	Annexin A5	0.40
Q9D8N0	Elongation factor 1-gamma	0.40
P63242	Eukaryotic translation initiation factor 5A-1	0.40
P19096	Fatty acid synthase	0.40
Q9Z2X1	Heterogeneous nuclear ribonucleoprotein F	0.40
Q9EPL8	Importin-7	0.40
Q9JHU9	Inositol-3-phosphate synthase 1	0.40
Q9CXY6	Interleukin enhancer-binding factor 2	0.40
Q99MN1	Lysine--tRNA ligase	0.40
O35286	Putative pre-mRNA-splicing factor ATP-dependent RNA helicase DHX15	0.40
P35486	Pyruvate dehydrogenase E1 component subunit alpha_ somatic form_ mitochondrial	0.40
P80318	T-complex protein 1 subunit gamma	0.40
Q6ZWM4	U6 snRNA-associated Sm-like protein LSm8	0.40
P14131	40S ribosomal protein S16	0.41
P62855	40S ribosomal protein S26	0.41
Q99JY9	Actin-related protein 3	0.41
Q99L45	Eukaryotic translation initiation factor 2 subunit 2	0.41
Q9R0N0	Galactokinase	0.41
P16045	Galectin-1	0.41
Q921M3-2	Isoform 2 of Splicing factor 3B subunit 3	0.41
Q9WU78	Programmed cell death 6-interacting protein	0.41
Q91V89	Protein Ppp2r5d	0.41
P52480	Pyruvate kinase PKM	0.41
P62315	Small nuclear ribonucleoprotein Sm D1	0.41

Q62261	Spectrin beta chain_ non-erythrocytic 1	0.41
Q99JB2	Stomatin-like protein 2_ mitochondrial	0.41
P05213	Tubulin alpha-1B chain	0.41
Q921H8	3-ketoacyl-CoA thiolase A_ peroxisomal	0.42
P62301	40S ribosomal protein S13	0.42
P60867	40S ribosomal protein S20	0.42
Q91V55	40S ribosomal protein S5	0.42
P47962	60S ribosomal protein L5	0.42
Q9DCD0	6-phosphogluconate dehydrogenase_ decarboxylating	0.42
Q8CGC7	Bifunctional glutamate/proline--tRNA ligase	0.42
Q91V12	Cytosolic acyl coenzyme A thioester hydrolase	0.42
Q8QZY1	Eukaryotic translation initiation factor 3 subunit L	0.42
Q05D44	Eukaryotic translation initiation factor 5B	0.42
P19157	Glutathione S-transferase P 1	0.42
P84104-2	Isoform Short of Serine/arginine-rich splicing factor 3	0.42
Q9QUR6	Prolyl endopeptidase	0.42
Q9R1P0	Proteasome subunit alpha type-4	0.42
P27773	Protein disulfide-isomerase A3	0.42
P97855	Ras GTPase-activating protein-binding protein 1	0.42
Q78PY7	Staphylococcal nuclease domain-containing protein 1	0.42
Q60864	Stress-induced-phosphoprotein 1	0.42
P68368	Tubulin alpha-4A chain	0.42
Q8BYA0	Tubulin-specific chaperone D	0.42
Q91YR1	Twinfilin-1	0.42
P61079	Ubiquitin-conjugating enzyme E2 D3	0.42
D3Z061	Ubiquitin-like modifier-activating enzyme 6	0.42
P62908	40S ribosomal protein S3	0.43
P27659	60S ribosomal protein L3	0.43
Q9D186	Apolipoprotein O	0.43
Q9CWJ9	Bifunctional purine biosynthesis protein PURH	0.43
P70372	ELAV-like protein 1	0.43
A2AN08-3	Isoform 3 of E3 ubiquitin-protein ligase UBR4	0.43
Q9Z204-4	Isoform 4 of Heterogeneous nuclear ribonucleoproteins C1/C2	0.43
G3UWG1	MCG115977	0.43
D3YU17	Nicalin	0.43
P27048	Small nuclear ribonucleoprotein-associated protein B	0.43
Q921L3	Transmembrane and coiled-coil domain-containing protein 1	0.43
O35900	U6 snRNA-associated Sm-like protein LSm2	0.43
Q02053	Ubiquitin-like modifier-activating enzyme 1	0.43
P62270	40S ribosomal protein S18	0.44
Q6ZWV3	60S ribosomal protein L10	0.44
A0A087WP24	Alpha/beta hydrolase domain-containing protein 14B (Fragment)	0.44
Q9DBG3	AP-2 complex subunit beta	0.44
Q9DAW9	Calponin-3	0.44
P70698	CTP synthase 1	0.44

O70378	ER membrane protein complex subunit 8	0.44
D3Z7P3	Glutaminase kidney isoform_ mitochondrial	0.44
Q8VEK3	Heterogeneous nuclear ribonucleoprotein U	0.44
P57776-2	Isoform 2 of Elongation factor 1-delta	0.44
Q9MD82	NADH-ubiquinone oxidoreductase chain 5	0.44
E9PWY9	Phenylalanine--tRNA ligase alpha subunit	0.44
Q61656	Probable ATP-dependent RNA helicase DDX5	0.44
Q91VI7	Ribonuclease inhibitor	0.44
P42669	Transcriptional activator protein Pur-alpha	0.44
Q5SVW9	Transmembrane emp24 domain-containing protein 4 (Fragment)	0.44
Q8C2E7	WASH complex subunit strumpellin	0.44
Q9D2R0	Acetoacetyl-CoA synthetase	0.45
Q9CVB6	Actin-related protein 2/3 complex subunit 2	0.45
P47738	Aldehyde dehydrogenase_ mitochondrial	0.45
Q5SVG5	AP complex subunit beta	0.45
Q91V92	ATP-citrate synthase	0.45
Q63829	COMM domain-containing protein 3	0.45
P68040	Guanine nucleotide-binding protein subunit beta-2-like 1	0.45
Q61425	Hydroxyacyl-coenzyme A dehydrogenase_ mitochondrial	0.45
Q9CPY7-2	Isoform 2 of Cytosol aminopeptidase	0.45
Q8K1M6-3	Isoform 3 of Dynamin-1-like protein	0.45
Q9CPQ3	Mitochondrial import receptor subunit TOM22 homolog	0.45
P50543	Protein S100-A11	0.45
P62317	Small nuclear ribonucleoprotein Sm D2	0.45
Q9EQH3	Vacuolar protein sorting-associated protein 35	0.45
P62192	26S protease regulatory subunit 4	0.46
P48962	ADP/ATP translocase 1	0.46
Q9CQ69	Cytochrome b-c1 complex subunit 8	0.46
D3YVN7	Elongation factor Tu	0.46
E9PVC6	Eukaryotic translation initiation factor 4 gamma 1	0.46
P47754	F-actin-capping protein subunit alpha-2	0.46
Q64152-2	Isoform 2 of Transcription factor BTF3	0.46
O35682	Myeloid-associated differentiation marker	0.46
P26883	Peptidyl-prolyl cis-trans isomerase FKBP1A	0.46
O55234	Proteasome subunit beta type-5	0.46
Q8VHN8	Protein syndesmos	0.46
O08583	THO complex subunit 4	0.46
Q9JK81	UPF0160 protein MYG1_ mitochondrial	0.46
B1AU25	Apoptosis-inducing factor 1_ mitochondrial	0.47
Q922B2	Aspartate--tRNA ligase_ cytoplasmic	0.47
Q64525	Histone H2B type 2-B	0.47
Q91VA7	Isocitrate dehydrogenase [NAD] subunit_ mitochondrial	0.47
P06151	L-lactate dehydrogenase A chain	0.47
Q61171	Peroxiredoxin-2	0.47
B2RXS4	Plexin-B2	0.47
O35129	Prohibitin-2	0.47

Q60692	Proteasome subunit beta type-6	0.47
Q80ZX0	Protein Sec24b	0.47
P62305	Small nuclear ribonucleoprotein E	0.47
D3Z7P2	Transmembrane protein 109 (Fragment)	0.47
Q99JI4	26S proteasome non-ATPase regulatory subunit 6	0.48
Q6ZWZ6	40S ribosomal protein S12	0.48
P63276	40S ribosomal protein S17	0.48
Q9D3D9	ATP synthase subunit delta_ mitochondrial	0.48
Q99KK7	Dipeptidyl peptidase 3	0.48
P62748	Hippocalcin-like protein 1	0.48
E9QKZ2	Importin-9	0.48
Q8BGJ5	MCG13402_ isoform CRA_a	0.48
Q99KE1	NAD-dependent malic enzyme_ mitochondrial	0.48
Q8BHN3	Neutral alpha-glucosidase AB	0.48
Q9QUM9	Proteasome subunit alpha type-6	0.48
Q99PT1	Rho GDP-dissociation inhibitor 1	0.48
Q5FWK3	Rho GTPase-activating protein 1	0.48
Q9D0K2	Succinyl-CoA:3-ketoacid coenzyme A transferase 1_ mitochondrial	0.48
Q3TW96	UDP-N-acetylhexosamine pyrophosphorylase-like protein 1	0.48
Q6ZWU9	40S ribosomal protein S27	0.49
P10852	4F2 cell-surface antigen heavy chain	0.49
Q9WTL7	Acyl-protein thioesterase 2	0.49
P51881	ADP/ATP translocase 2	0.49
O08553	Dihydropyrimidinase-related protein 2	0.49
Q9D7X3	Dual specificity protein phosphatase 3	0.49
Q99KJ8	Dynactin subunit 2	0.49
Q8BWY3	Eukaryotic peptide chain release factor subunit 1	0.49
Q8BMJ3	Eukaryotic translation initiation factor 1A_ X-chromosomal	0.49
Q9Z0N1	Eukaryotic translation initiation factor 2 subunit 3_ X-linked	0.49
Q3U0V1	Far upstream element-binding protein 2	0.49
P62849-2	Isoform 2 of 40S ribosomal protein S24	0.49
Q9D0M3-2	Isoform 2 of Cytochrome c1_ heme protein_ mitochondrial	0.49
P03899	NADH-ubiquinone oxidoreductase chain 3	0.49
Q501J6	Probable ATP-dependent RNA helicase DDX17	0.49
D3Z0A2	Protein arginine N-methyltransferase 1	0.49
P14069	Protein S100-A6	0.49
P61027	Ras-related protein Rab-10	0.49
P99024	Tubulin beta-5 chain	0.49
Q99N15	17beta-hydroxysteroid dehydrogenase type 10/short chain L-3-hydroxyacyl-CoA dehydrogenase	0.50
Q60597	2-oxoglutarate dehydrogenase_ mitochondrial	0.50
Q5EG47	5'-AMP-activated protein kinase catalytic subunit alpha-1	0.50
P60710	Actin_ cytoplasmic 1	0.50
Q7TPR4	Alpha-actinin-1	0.50
D3YW48	Calpain small subunit 1 (Fragment)	0.50

Q9CZU6	Citrate synthase_ mitochondrial	0.50
Q6ZQ38	Cullin-associated NEDD8-dissociated protein 1	0.50
P58252	Elongation factor 2	0.50
G3XA10	Heterogeneous nuclear ribonucleoprotein U_ isoform CRA_b	0.50
Q9JKR6	Hypoxia up-regulated protein 1	0.50
Q8CHK3	Lysophospholipid acyltransferase 7	0.50
Q69ZN7	Myoferlin	0.50
Q9DCT2	NADH dehydrogenase [ubiquinone] iron-sulfur protein 3_ mitochondrial	0.50
O70400	PDZ and LIM domain protein 1	0.50
Q11011	Puromycin-sensitive aminopeptidase	0.50
P20108	Thioredoxin-dependent peroxide reductase_ mitochondrial	0.50
Q9WVA4	Transgelin-2	0.50
P62274	40S ribosomal protein S29	0.51
P61164	Alpha-centractin	0.51
Q64133	Amine oxidase [flavin-containing] A	0.51
F8WHP8	ATP synthase subunit f_ mitochondrial	0.51
Q91VR5	ATP-dependent RNA helicase DDX1	0.51
P56380	Bis(5'-nucleosyl)-tetraphosphatase [asymmetrical]	0.51
Q8BH59	Calcium-binding mitochondrial carrier protein Aralar1	0.51
Q01149	Collagen alpha-2(I) chain	0.51
Q9CZ04	COP9 signalosome complex subunit 7a	0.51
Q9DB77	Cytochrome b-c1 complex subunit 2_ mitochondrial	0.51
Q7JCZ1	Cytochrome c oxidase subunit 2	0.51
P10630	Eukaryotic initiation factor 4A-II	0.51
Q3UEB3-3	Isoform 3 of Poly(U)-binding-splicing factor PUF60	0.51
Q61768	Kinesin-1 heavy chain	0.51
Q9EQK5	Major vault protein	0.51
P49722	Proteasome subunit alpha type-2	0.51
O70435	Proteasome subunit alpha type-3	0.51
E9PZ69	Transmembrane 9 superfamily member 2	0.51
Q922F4	Tubulin beta-6 chain	0.51
P68037	Ubiquitin-conjugating enzyme E2 L3	0.51
Q9R0Y5	Adenylate kinase isoenzyme 1	0.52
B0R091	Calcineurin B homologous protein 1	0.52
O88587	Catechol O-methyltransferase	0.52
Q8VBZ3	Cleft lip and palate transmembrane protein 1 homolog	0.52
Q9QZE5	Coatomer subunit gamma-1	0.52
Q9WVK4	EH domain-containing protein 1	0.52
O35737	Heterogeneous nuclear ribonucleoprotein H	0.52
Q9QYB1	Chloride intracellular channel protein 4	0.52
Q60766-2	Isoform 2 of Immunity-related GTPase family M protein 1	0.52
Q99LB6-2	Isoform 2 of Methionine adenosyltransferase 2 subunit beta	0.52
P28660-2	Isoform 2 of Nck-associated protein 1	0.52
Q8BJU0-2	Isoform 2 of Small glutamine-rich tetratricopeptide repeat-containing protein alpha	0.52
P08249	Malate dehydrogenase_ mitochondrial	0.52

P26041	Moesin	0.52
Q9DCS9	NADH dehydrogenase [ubiquinone] 1 beta subcomplex subunit 10	0.52
Q9D8S4	Oligoribonuclease_ mitochondrial	0.52
P50396	Rab GDP dissociation inhibitor alpha	0.52
Q9CQD1	Ras-related protein Rab-5A	0.52
D3YWT0	Signal peptidase I	0.52
Q9JHJ0	Tropomodulin-3	0.52
P23927	Alpha-crystallin B chain	0.53
Q3TWV4	AP-2 complex subunit mu	0.53
P05202	Aspartate aminotransferase_ mitochondrial	0.53
Q9D2G2	Dihydrolipoyllysine-residue succinyltransferase component of 2-oxoglutarate dehydrogenase complex_ mitochondrial	0.53
P61804	Dolichyl-diphosphooligosaccharide--protein glycosyltransferase subunit DAD1	0.53
P42125	Enoyl-CoA delta isomerase 1_ mitochondrial	0.53
P70333	Heterogeneous nuclear ribonucleoprotein H2	0.53
P97823-2	Isoform 2 of Acyl-protein thioesterase 1	0.53
P37889-2	Isoform 2 of Fibulin-2	0.53
A0A087WNW3	Kinectin	0.53
A0A0A6YW67	MCG23377_ isoform CRA_b	0.53
P29595	NEDD8	0.53
Q8BH04	Phosphoenolpyruvate carboxykinase [GTP]_ mitochondrial	0.53
D3Z645	Vacuolar protein sorting-associated protein 29	0.53
B1AR28	Very long-chain-specific acyl-CoA dehydrogenase_ mitochondrial	0.53
P62814	V-type proton ATPase subunit B_ brain isoform	0.53
O88342	WD repeat-containing protein 1	0.53
Q9Z2W0	Aspartyl aminopeptidase	0.54
O35350	Calpain-1 catalytic subunit	0.54
P10605	Cathepsin B	0.54
P19783	Cytochrome c oxidase subunit 4 isoform 1_ mitochondrial	0.54
Q61753	D-3-phosphoglycerate dehydrogenase	0.54
H3BLR8	Diphosphoinositol polyphosphate phosphohydrolase 1	0.54
A0A087WR50	Fibronectin	0.54
P14602	Heat shock protein beta-1	0.54
Q8C3X8	Lipase maturation factor 2	0.54
Q9CQ75	NADH dehydrogenase [ubiquinone] 1 alpha subcomplex subunit 2	0.54
Q8K3J1	NADH dehydrogenase [ubiquinone] iron-sulfur protein 8_ mitochondrial	0.54
Q8CI51	PDZ and LIM domain protein 5	0.54
P35700	Peroxiredoxin-1	0.54
D3Z2X5	Platelet-activating factor acetylhydrolase IB subunit gamma (Fragment)	0.54
Q8VE70	Programmed cell death protein 10	0.54
Q9R1P1	Proteasome subunit beta type-3	0.54

P08207	Protein S100-A10	0.54
Q921E2	Ras-related protein Rab-31	0.54
P51150	Ras-related protein Rab-7a	0.54
Q9CYN2	Signal peptidase complex subunit 2	0.54
Q61263	Sterol O-acyltransferase 1	0.54
Q9CQW1	Synaptobrevin homolog YKT6	0.54
A0A087WQE6	Transcription elongation factor B polypeptide 1 (Fragment)	0.54
Q9Z1Q9	Valine--tRNA ligase	0.54
G3XA25	Acetyl-CoA acetyltransferase_ cytosolic	0.55
P61161	Actin-related protein 2	0.55
P61750	ADP-ribosylation factor 4	0.55
Q9QWR8	Alpha-N-acetylgalactosaminidase	0.55
Q8VCQ8	Caldesmon 1	0.55
Q8BXC6	COMM domain-containing protein 2	0.55
Q8R395	COMM domain-containing protein 5	0.55
Q8R1B4	Eukaryotic translation initiation factor 3 subunit C	0.55
Q00612	Glucose-6-phosphate 1-dehydrogenase X	0.55
P26443	Glutamate dehydrogenase 1_ mitochondrial	0.55
Q62470	Integrin alpha-3	0.55
P68254-2	Isoform 2 of 14-3-3 protein theta	0.55
Q9DCF9-2	Isoform 2 of Translocon-associated protein subunit gamma	0.55
Q7M739	Nuclear pore complex-associated intranuclear coiled-coil protein TPR	0.55
Q8VEM8	Phosphate carrier protein_ mitochondrial	0.55
Q9R0E1	Procollagen-lysine_2-oxoglutarate 5-dioxygenase 3	0.55
O88952	Protein lin-7 homolog C	0.55
P46638	Ras-related protein Rab-11B	0.55
O55131	Septin-7	0.55
Q91V61	Sideroflexin-3	0.55
Q9QUI0	Transforming protein RhoA	0.55
Q9QZE7	Translin-associated protein X	0.55
Q9CY50	Translocon-associated protein subunit alpha	0.55
Q8BMS1	Trifunctional enzyme subunit alpha_ mitochondrial	0.55
Q8VDJ3	Vigilin	0.55
P20152	Vimentin	0.55
Q99KI0	Aconitate hydratase_ mitochondrial	0.56
P50247	Adenosylhomocysteinase	0.56
Q9JII6	Alcohol dehydrogenase [NADP(+)]	0.56
Q07076	Annexin A7	0.56
Q8R180	ERO1-like protein alpha	0.56
P13020-2	Isoform 2 of Gelsolin	0.56
Q9DCN2-2	Isoform 2 of NADH-cytochrome b5 reductase 3	0.56
Q6ZWQ9	MCG5400	0.56
Q9CQ54	NADH dehydrogenase [ubiquinone] 1 subunit C2	0.56
Q99PV0	Pre-mRNA-processing-splicing factor 8	0.56
P08003	Protein disulfide-isomerase A4	0.56
O89086	RNA-binding protein 3	0.56

P42208	Septin-2	0.56
Q6PDM2	Serine/arginine-rich splicing factor 1	0.56
Q9CWK8	Sorting nexin-2	0.56
P61089	Ubiquitin-conjugating enzyme E2 N	0.56
P62889	60S ribosomal protein L30	0.57
Q9ET22	Dipeptidyl peptidase 2	0.57
Q8CFQ9	Fusion_ derived from t(1216) malignant liposarcoma (Human)	0.57
Q9DBF1-2	Isoform 2 of Alpha-aminoadipic semialdehyde dehydrogenase	0.57
Q8K310	Matrin-3	0.57
Q9ERS2	NADH dehydrogenase [ubiquinone] 1 alpha subcomplex subunit 13	0.57
Q9DBG5	Perilipin-3	0.57
D3Z074	Protein diaphanous homolog 1	0.57
Q9WVE8	Protein kinase C and casein kinase substrate in neurons protein 2	0.57
P63001	Ras-related C3 botulinum toxin substrate 1	0.57
Q9DBH5	Vesicular integral-membrane protein VIP36	0.57
P50518	V-type proton ATPase subunit E 1	0.57
Q8BG32	26S proteasome non-ATPase regulatory subunit 11	0.58
P14685	26S proteasome non-ATPase regulatory subunit 3	0.58
P35762	CD81 antigen	0.58
Q8BML9	Glutaminyl-tRNA synthetase	0.58
Q9Z1Q5	Chloride intracellular channel protein 1	0.58
P51174	Long-chain specific acyl-CoA dehydrogenase_ mitochondrial	0.58
P34884	Macrophage migration inhibitory factor	0.58
Q9D7B7	Probable glutathione peroxidase 8	0.58
P62137	Serine/threonine-protein phosphatase PP1-alpha catalytic subunit	0.58
P54775	26S protease regulatory subunit 6B	0.59
P57780	Alpha-actinin-4	0.59
Q9Z1T1	AP-3 complex subunit beta-1	0.59
P34914	Bifunctional epoxide hydrolase 2	0.59
O55029	Coatomer subunit beta'	0.59
P01027	Complement C3	0.59
Q62425	Cytochrome c oxidase subunit NDUFA4	0.59
Q9JHU4	Cytoplasmic dynein 1 heavy chain 1	0.59
P29387	Guanine nucleotide-binding protein subunit beta-4	0.59
Q9DBJ1	Phosphoglycerate mutase 1	0.59
P07091	Protein S100-A4	0.59
Q9D051	Pyruvate dehydrogenase E1 component subunit beta_ mitochondrial	0.59
P26043	Radixin	0.59
P35293	Ras-related protein Rab-18	0.59
Q99JR1	Sideroflexin-1	0.59
P62281	40S ribosomal protein S11	0.60

P51410	60S ribosomal protein L9	0.60
P24668	Cation-dependent mannose-6-phosphate receptor	0.60
Q4KML4	Costars family protein ABRACL	0.60
Q9DCT8	Cysteine-rich protein 2	0.60
Q8BH64	EH domain-containing protein 2	0.60
Q9DCW4	Electron transfer flavoprotein subunit beta	0.60
Q9WTP7	GTP:AMP phosphotransferase AK3_ mitochondrial	0.60
P08752	Guanine nucleotide-binding protein G(i) subunit alpha-2	0.60
Q60973	Histone-binding protein RBBP7	0.60
P14152	Malate dehydrogenase_ cytoplasmic	0.60
Q9CQC7	NADH dehydrogenase [ubiquinone] 1 beta subcomplex subunit 4	0.60
P57716	Nicastrin	0.60
Q9CQ65	S-methyl-5'-thioadenosine phosphorylase	0.60
Q64310	Surfeit locus protein 4	0.60
Q62186	Translocon-associated protein subunit delta	0.60
Q7TMM9	Tubulin beta-2A chain	0.60
O70475	UDP-glucose 6-dehydrogenase	0.60
Q6ZQM8	UDP-glucuronosyltransferase 1-7C	0.60
Q9CY27	Very-long-chain enoyl-CoA reductase	0.60
P56480	ATP synthase subunit beta_ mitochondrial	0.61
Q8VDW0	ATP-dependent RNA helicase DDX39A	0.61
O08529	Calpain-2 catalytic subunit	0.61
Q9D379	Epoxide hydrolase 1	0.61
Q3U7R1	Extended synaptotagmin-1	0.61
Q8CI94	Glycogen phosphorylase_ brain form	0.61
O88844	Isocitrate dehydrogenase [NADP] cytoplasmic	0.61
Q9D7S7-2	Isoform 2 of 60S ribosomal protein L22-like 1	0.61
Q05BC3-2	Isoform 2 of Echinoderm microtubule-associated protein-like 1	0.61
Q6ZQI3	Malectin	0.61
Q80UU9	Membrane-associated progesterone receptor component 2	0.61
O35683	NADH dehydrogenase [ubiquinone] 1 alpha subcomplex subunit 1	0.61
Q99K48	Non-POU domain-containing octamer-binding protein	0.61
P35282	Ras-related protein Rab-21	0.61
Q91ZR1	Ras-related protein Rab-4B	0.61
Q07417	Short-chain specific acyl-CoA dehydrogenase_ mitochondrial	0.61
P62983	Ubiquitin-40S ribosomal protein S27a	0.61
O35593	26S proteasome non-ATPase regulatory subunit 14	0.62
P68134	Actin_ alpha skeletal muscle	0.62
P17427	AP-2 complex subunit alpha-2	0.62
Q02248	Catenin beta-1	0.62
P60766	Cell division control protein 42 homolog	0.62
Q9WUM4	Coronin-1C	0.62
Q91YQ5	Dolichyl-diphosphooligosaccharide--protein glycosyltransferase subunit 1	0.62

Q8BH95	Enoyl-CoA hydratase_ mitochondrial	0.62
P21107-2	Isoform 2 of Tropomyosin alpha-3 chain	0.62
Q80SW1	Putative adenosylhomocysteinase 2	0.62
Q9D031	Ras suppressor protein 1	0.62
P10833	Ras-related protein R-Ras	0.62
Q9CQA3	Succinate dehydrogenase [ubiquinone] iron-sulfur subunit_ mitochondrial	0.62
P68510	14-3-3 protein eta	0.63
A1BN54	Alpha actinin 1a	0.63
P27046	Alpha-mannosidase 2	0.63
Q9CZ13	Cytochrome b-c1 complex subunit 1_ mitochondrial	0.63
Q9R0P5	Destrin	0.63
Q3THW5	Histone H2A.V	0.63
Q61598-2	Isoform 2 of Rab GDP dissociation inhibitor beta	0.63
Q6P069-2	Isoform 2 of Sorcin	0.63
P11881-8	Isoform 8 of Inositol 1_4_5-trisphosphate receptor type 1	0.63
Q61033	Lamina-associated polypeptide 2_ isoforms alpha/zeta	0.63
Q7TMF3	NADH dehydrogenase [ubiquinone] 1 alpha subcomplex subunit 12	0.63
F8VPK5	Rho-associated protein kinase	0.63
Q64105	Sepiapterin reductase	0.63
P09671	Superoxide dismutase [Mn]_ mitochondrial	0.63
O08547	Vesicle-trafficking protein SEC22b	0.63
P23780	Beta-galactosidase	0.64
Q9CQI6	Coactosin-like protein	0.64
Q9CPQ1	Cytochrome c oxidase subunit 6C	0.64
F7A6H4	E3 ubiquitin-protein ligase RNF213	0.64
Q9CPX4	Ferritin	0.64
E9Q1S3	Heterogeneous nuclear ribonucleoprotein K (Fragment)	0.64
P24527	Leukotriene A-4 hydrolase	0.64
Q7TPV4	Myb-binding protein 1A	0.64
Q91V41	Ras-related protein Rab-14	0.64
A2AVJ7	Ribosome-binding protein 1	0.64
E9PXX7	Thioredoxin domain-containing protein 5	0.64
Q99JY0	Trifunctional enzyme subunit beta_ mitochondrial	0.64
Q9WTI7	Unconventional myosin-Ic	0.64
Q9DCR2	AP-3 complex subunit sigma-1	0.65
P97742	Carnitine O-palmitoyltransferase 1_ liver isoform	0.65
Q8CIE6	Coatomer subunit alpha	0.65
Q8VHX6	Filamin-C	0.65
F6SAC3	Glucose-6-phosphate isomerase	0.65
Q61696	Heat shock 70 kDa protein 1A	0.65
Q3UPL0-2	Isoform 2 of Protein transport protein Sec31A	0.65
P85094	Isochorismatase domain-containing protein 2A_ mitochondrial	0.65
P06795	Multidrug resistance protein 1B	0.65
P29341	Polyadenylate-binding protein 1	0.65
Q922R8	Protein disulfide-isomerase A6	0.65

Q3TCN2	Putative phospholipase B-like 2	0.65
D3Z3A9	Reticulocalbin-3 (Fragment)	0.65
P62717	60S ribosomal protein L18a	0.66
F8WIT2	Annexin	0.66
O35841	Apoptosis inhibitor 5	0.66
G5E850	Cytochrome b-5_ isoform CRA_a	0.66
G3X9L6	MCG55033	0.66
P80313	T-complex protein 1 subunit eta	0.66
P58871	182 kDa tankyrase-1-binding protein	0.67
Q9JIF7	Coatomer subunit beta	0.67
P62880	Guanine nucleotide-binding protein G(I)/G(S)/G(T) subunit beta-2	0.67
P70699	Lysosomal alpha-glucosidase	0.67
Q9CQS8	Protein transport protein Sec61 subunit beta	0.67
Q9R0M6	Ras-related protein Rab-9A	0.67
Q8VIJ6	Splicing factor_ proline- and glutamine-rich	0.67
Q8JZU2	Tricarboxylate transport protein_ mitochondrial	0.67
Q91ZJ5	UTP--glucose-1-phosphate uridylyltransferase	0.67
P46935	E3 ubiquitin-protein ligase NEDD4	0.68
P39054-2	Isoform 2 of Dynamin-2	0.68
Q9R1P3	Proteasome subunit beta type-2	0.68
P63024	Vesicle-associated membrane protein 3	0.68
G3X9U9	Fission 1 (Mitochondrial outer membrane) homolog (Yeast)_ isoform CRA_c	0.69
O88322	Nidogen-2	0.69
Q61316	Heat shock 70 kDa protein 4	0.70
A2AKI5	Integrin alpha-V	0.70
Q8BU30	Isoleucine--tRNA ligase_ cytoplasmic	0.70
P59383	Leucine-rich repeat neuronal protein 4	0.70
Q6A099	MKIAA0248 protein (Fragment)	0.70
P08228	Superoxide dismutase [Cu-Zn]	0.70
A2AKU9	ATP synthase subunit gamma	0.71
Q3UF75	Alpha-parvin	0.72
F8WHM5	Golgi apparatus protein 1 (Fragment)	0.72
Q62426	Cystatin-B	0.73
Q9ERN0	Secretory carrier-associated membrane protein 2	0.73

Table S4. Differentially expressed proteins during transmigration of MIM-RT cells.

Uniprot ID	Description	Expression Ratio Sample/Control
Q922R8	Protein disulfide-isomerase A6	1.36
Q80ZJ2	Impa1 protein	1.35
P19157	Glutathione S-transferase P 1	1.24
P62911	60S ribosomal protein L32	0.50
Q543K9	Purine nucleoside phosphorylase	0.51
P61222	ATP-binding cassette sub-family E member 1	0.54
P26443	Glutamate dehydrogenase 1_ mitochondrial	0.54
P14901	Heme oxygenase 1	0.65
P63028	Translationally-controlled tumor protein	0.65
P07356	Annexin A2	0.66
D3YWF6	Ubiquitin thioesterase OTUB1	0.66
E9Q616	Protein Ahnak	0.67
Q60973	Histone-binding protein RBBP7	0.68
P55264-2	Isoform Short of Adenosine kinase	0.68
Q99LB4	Capping protein (Actin filament)_ gelsolin-like	0.69
Q9Z1Q5	Chloride intracellular channel protein 1	0.70
Q9CPU0	Lactoylglutathione lyase	0.70
Q8VHX6	Filamin-C	0.72
Q7TPV4	Myb-binding protein 1A	0.72
Q6GT24	Peroxiredoxin 6	0.72
P10639	Thioredoxin	0.74
P47962	60S ribosomal protein L5	0.75
Q9JHU4	Cytoplasmic dynein 1 heavy chain 1	0.75
P57776	Elongation factor 1-delta	0.75
P57776-2	Isoform 2 of Elongation factor 1-delta	0.75
Z4YKM2	CDGSH iron-sulfur domain-containing protein 3_ mitochondrial	0.76
Q91V41	Ras-related protein Rab-14	0.76
P26638	Serine--tRNA ligase_ cytoplasmic	0.76
Q9DB20	ATP synthase subunit O_ mitochondrial	0.77
Q9CXW3	Calcyclin-binding protein	0.77
P14211	Calreticulin	0.77
Q9D8N0	Elongation factor 1-gamma	0.77
P54071	Isocitrate dehydrogenase [NADP]_ mitochondrial	0.77
Q8BP67	60S ribosomal protein L24	0.80
Q64525	Histone H2B type 2-B	0.80
P05202	Aspartate aminotransferase_ mitochondrial	0.83
